# Diffuse large B‐cell lymphoma initially presenting with peritoneal dissemination

**DOI:** 10.1002/ccr3.5191

**Published:** 2021-12-11

**Authors:** Risa Hirata, Masaki Tago, Yoshinori Tokushima

**Affiliations:** ^1^ Department of General Medicine Saga University Hospital Saga Japan

**Keywords:** diffuse large B‐cell lymphoma, peritoneal dissemination, ultrasound‐guided biopsy

## Abstract

A 62‐year‐old woman with a severely distended abdomen and no palpable superficial lymph nodes visited the hospital. Computed tomography with contrast enhancement revealed multiple fused and homogeneously contrasting masses filling the abdominal cavity. She was diagnosed with diffuse large B‐cell lymphoma by ultrasound‐guided needle biopsy performed on admission.

## Graphical abstract

Malignant lymphoma can be a cause of peritoneal dissemination of unknown primary lesions. Because malignant lymphomas which present with peritoneal dissemination are usually highly malignant, they should be diagnosed immediately.

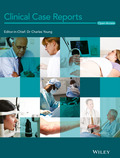

## 1 CASE

**FIGURE 1:**
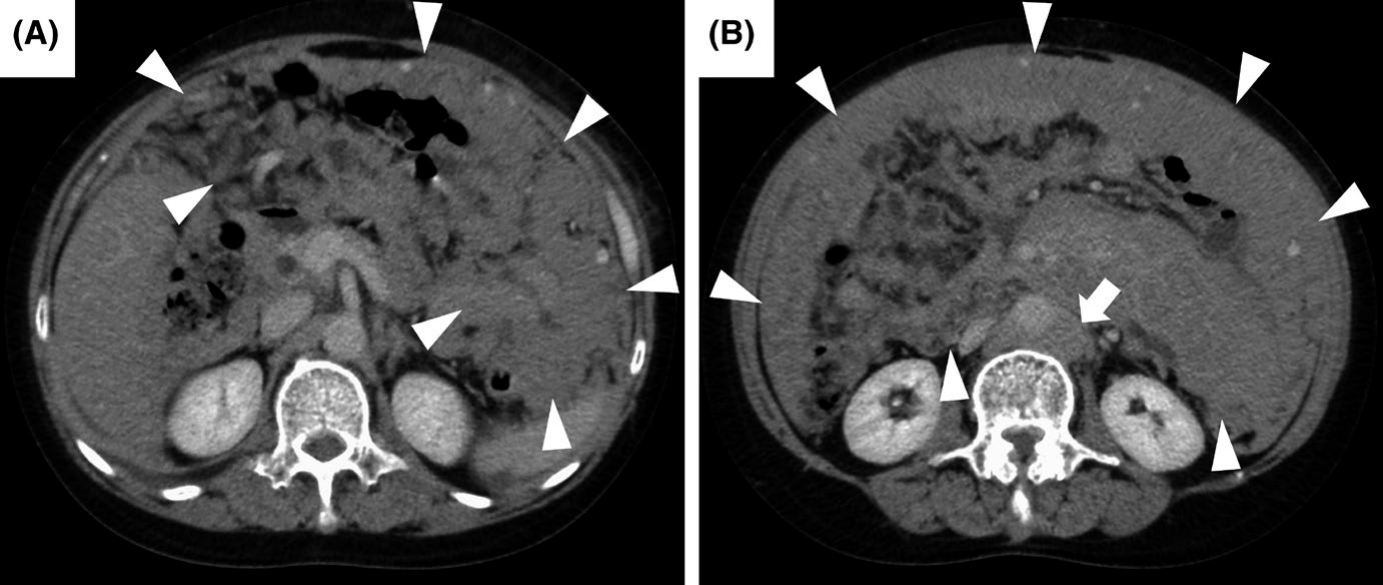
Chest and abdominal computed tomography with contrast enhancement. Chest and abdominal computed tomography with contrast enhancement revealed multiple fused and homogeneously contrasting masses filling the abdominal cavity (A, B: arrowheads) and para‐aortic lymph nodes (B: arrow)

A 62‐year‐old woman presented with a 3‐month history of anorexia and a 1‐month history of abdominal fullness and night sweats. On admission, physical examination revealed tachycardia, tachypnea, severe abdominal distension, and no palpable superficial lymph nodes. Laboratory examination showed a normal peripheral blood smear, lactate dehydrogenase concentration of 1,215 U/L, and soluble interleukin‐2 receptor concentration of 13,207 U/mL. Chest and abdominal contrast‐enhanced computed tomography revealed multiple fused masses without primary lesions. The masses were homogeneously contrasting and filling the abdominal cavity (Figure 1). Upper and lower gastrointestinal endoscopy showed no gastrointestinal cancer. She was diagnosed with diffuse large B‐cell lymphoma by ultrasound‐guided needle biopsy performed on admission, and chemotherapy was started on Day 3.

Malignant lymphoma rarely causes peritoneal dissemination because the peritoneum has no lymphatic tissue; however, dissemination is possible.^1^ Metastasis by solid cancer of the ovary, colon, or pancreas is a well‐known cause of peritoneal dissemination.^1^ Peritoneal dissemination due to malignant lymphoma, which can be highly malignant,^1^ presents with ascites, enlarged lymph nodes, bulky homogeneous masses, and diffuse thickness of the mesentery and peritoneum.^2^ Therefore, in patients with peritoneal dissemination, malignant lymphoma should be suspected as a cause of peritoneal dissemination after ruling out those solid cancers.

## CONFLICT OF INTEREST

The authors state that they have no conflict of interest.

## AUTHOR CONTRIBUTIONS

RH: was involved in the literature search, study conception, drafting of the manuscript, and clinical care of the patient. MT: was involved in the literature search, study conception, and drafting and revision of the manuscript. YT: was involved in the literature search, drafting of the manuscript, and clinical care of the patient.

## ETHICAL APPROVAL

This manuscript conforms to the provisions of the Declaration of Helsinki in 1995 (as revised in Brazil 2013).

## CONSENT

Written informed consent was obtained from the patient to publish this report in accordance with the journal's patient consent policy.

## Data Availability

The data that support the findings of this study are available from the corresponding author upon reasonable request.
